# Relative age selection bias does not translate into a playing-time advantage: evidence from Italian Serie A football

**DOI:** 10.3389/fpsyg.2025.1733277

**Published:** 2026-01-05

**Authors:** Paolo Riccardo Brustio, Alexandru Nicolae Ungureanu, Luca Beratto, Damiano Li Volsi, Corrado Lupo

**Affiliations:** 1 Department of Clinical and Biological Sciences, University of Turin, Turin, Italy; 2 Neuromuscular Function Research Group, School of Exercise & Sport Sciences, University of Turin, Turin, Italy; 3 Department of Life Sciences and System Biology, University of Turin, Turin, Italy; 4 Department of Medical Sciences, University of Turin, Turin, Italy

**Keywords:** RAE, soccer, selection bias, match-related performance, talent identification

## Abstract

**Introduction:**

The Relative Age Effects (RAEs) represent a bias in talent identification and selection processes, favouring athletes born earlier in the selection year. This study aimed to (i) quantify the prevalence and magnitude of the RAEs in Italian elite football (Serie A), considering age categories, playing positions, and team ranking, and (ii) examine whether RAEs are associated with selection- and participation-related outcomes (i.e., match appearances, total playing time, and consistency of match participation).

**Methods:**

Data from 789 Serie A players were analysed.

**Results:**

Q1 players were overrepresented compared with Q4 players (OR=1.9 [1.5–2.6]). RAEs were more pronounced in younger players (OR=2.1 [1.4–3.2]) and more evident among goalkeepers (OR=4.4 [1.8–10.6]) followed by forwards (OR=2.0 [1.0–3.8]), midfielders (OR=1.9 [1.1–3.1]) and defenders (OR=1.6 [1.0–2.4]). Moreover, RAEs were stronger in lower-tier teams (OR=2.5 [1.4–4.5]) than in top-tier teams (OR=1.5 [0.8–2.7]). No differences were observed between quartiles in selection- and participation-related outcomes.

**Discussion:**

RAEs persist in Serie A rosters but appear to operate primarily as a selection-level bias within talent development and squad selection, affecting which players reach and remain at the elite level, rather than influencing coaches’ decisions regarding match participation once players are part of the professional environment.

## 1 Introduction

Relative age effects (RAEs) are a well-known phenomenon in both individual and team sports, reflecting the advantages and disadvantages generated by the interaction between chronological age and fixed annual cut-off dates (Cobley et al., 2009; Williams et al., 2020; Smith et al., 2018). The majority of sports federations adopt a fixed cut-off date (typically 1 January) to group youth players, aiming to ensure fairness in competition. Nevertheless, this grouping system inadvertently introduces age-related bias, particularly at the youth level, where the relative age gap may reach 12 or even 24 months in single-year or biennial cohorts, respectively. These relative age differences may confer advantages to Q1 players in their anthropometric, physiological, and psychosocial development, often translating into superior performance compared to their Q4 peers (Cobley et al., 2009). Consequently, relatively older players (Q1) are more likely—consciously or unconsciously—to be selected by coaches and scouts, whereas relatively younger players (Q4) are comparatively disadvantaged. Early selection in sports provide valuable developmental opportunities such as greater access to coaching, competition, facilities, and specialist support. However, these advantages may further confound identification and selection processes, ultimately hindering the transition from youth to senior levels.

Soccer is a fundamental example of this phenomenon, with RAEs consistently reported across different countries and competitive levels (Bozděch et al., 2023). The prevalence and magnitude of these effects depend on several moderating factors, including sex, age group, competition level, playing position, and broader sociocultural influences (i.e., attraction level, country, depth of competition, and historical period). Generally, RAEs are more pronounced among male players than female players, likely due to differences in participation rates, selection depth, and sociocultural expectations (Cobley et al., 2009; Bozděch et al., 2023; Brustio et al., 2022, 2024; Smith and Weir, 2020). Moreover, research has shown that the strongest asymmetries in favor of Q1 players occur in youth academies but diminish as players age (Doncaster et al., 2020). For example, in the FC Barcelona academy, the odds ratio (OR) of being selected for Q1 players was approximately 18 times higher than for Q4 players at the U10 level. This disparity declined to about nine at U12 seven at U18 (Doncaster et al., 2020). This trend remains evident even as competition increases. For example, a similar pattern was observed at a top-level Italian professional club, where the odds of being selected for Q1 players were approximately 10, 8, 4, 4, and 2 in the U15, U16, U17, U19, and Senior categories, respectively (Brustio et al., 2018). Similarly, recent data demonstrated skewed birthdate distributions favoring Q1 players at U17, U19, and U21 national team rosters (OR range 5.9–2.2) across multiple European countries (i.e., France, Germany, England, Italy, and Spain) (Brustio et al., 2024).

In addition to selection prevalence, a large body of research has examined RAEs in relation to physical and fitness characteristics in youth players, which can be considered indirect indicators of performance potential. From a physical -capacity perspective, several studies have shown that Q1 football players often display physical advantages in body size, strength, sprint speed, jumping ability, and aerobic capacity, although these differences are context-dependent, may reverse in older age groups, and tend to diminish with maturation (Deprez et al., 2012; Peña-González et al., 2021; Rađa et al., 2025). For instance, in the Italian context, Q1 players showed more favorable anthropometrics and better 15-m sprint and RSA performance in U12, U13, and U14 youth categories (Toselli et al., 2022). These findings were confirmed by Rađa et al. (2025) who reported that Q1 Croatian players were, on average, 5–9-cm taller and 11–17% heavier than their relatively younger peers, and they outperformed Q4 players in standing long jump (>5–7%), medicine ball throw (>13–17%), and sprinting tests (>2–3%), as well as in kicking tasks with both legs, but not in ball-handling skills such as slalom or zig-zag dribbling (Rađa et al., 2025). In contrast, Bezuglov et al. (2023) found a pronounced RAE (Q1 = 43.5% vs. Q4 = 9.6%) in male football players (aged 12–17) from Russian football academies, but no significant differences in strength, speed, or football-specific skills between the quartiles were observed. Similarly, physical performance measures (i.e., countermovement jump, 30-m sprint, *T*-test, and Yo–Yo endurance run) did not differ between Q1 and Q4 players in other settings (Deprez et al., 2012; Peña-González et al., 2021). In the context of English youth (U11 and U13), no differences were observed in functional capacities (e.g., shuttle run, countermovement jump, sprint, Yo–Yo endurance run), football skills (e.g., ball control, dribbling speed, passing, and shooting), or goal orientation (e.g., task and ego) across different birth quartiles (Figueiredo et al., 2019). Conversely, in older age groups, studies have reported the opposite trend, with Q4 players outperforming Q1 players in selected performance tests (Skorski et al., 2016; Patel et al., 2020). Overall, this literature suggests that RAEs in youth may reflect, at least in part, differences in physical maturation and fitness, which can serve as indirect markers of performance potential. However, it remains unclear whether such advantages persist and translate into competitive outcomes at later stages.

In adult professional football, studies of RAEs can therefore be interpreted as assessments of whether these early developmental asymmetries leave residual effects on who ultimately reaches and remains at the elite level. Although the magnitude of RAEs tends to decrease with age, the findings in adult professional contexts are less consistent. In some elite settings, residual asymmetries (i.e., knock-on effects) remain evident, particularly in highly competitive leagues, whereas in others, the effects disappear (Brustio et al., 2024; Boccia et al., 2023; Andrew et al., 2022). In the Italian context, previous research has shown that RAEs remain evident at the senior level, both in national teams (Boccia et al., 2023) and in the major domestic league (Serie A) (Brustio et al., 2018; Lupo et al., 2019).

In addition to documenting prevalence, a central question in adult and late-development cohorts is whether birth quartile asymmetries translate into advantages in selection- and participation-related outcomes across the youth-to-senior pathway. While numerous studies have quantified RAEs in football, fewer have investigated whether birthdate asymmetries translate into competitive advantages such as increased playing time, greater match involvement, or superior performance statistics. Emerging evidence suggests that birthdate can influence selection and subsequent career progression (Brustio et al., 2024; Boccia et al., 2023), but findings linking RAEs to selection- and participation-related outcomes in football are inconsistent and often contradictory.

At the team level, significant associations have been reported between RAEs and final team rankings (Augste and Lames, 2011; Fritzler, 2024), as well as individual success indicators (Fritzler, 2024). However, in a match-related analysis of Belgian senior football, Vaeyens et al. (2005) showed that although relatively older players were over-represented and appeared to receive greater overall opportunities (approximately 34% for Q1 and 19% for Q4 of match selection and minutes played), birth quartile did not translate into between-quartile differences in average selections or minutes played. Consistently, among Norwegian U20 players, despite clear RAEs in roster composition, birth quartile did not consistently predict playing time across seasons (Sæther, 2016). Similar findings emerged in a longitudinal analysis following players from U9 to senior level at Real Club Deportivo de la Coruña, where no differences in minutes played were found between birth quartiles (Rodríguez-Lorenzo and Martín-Acero, 2019).

Taken together, these results do not clarify whether these selection biases automatically translate into more minutes on the pitch or greater competitive involvement. This underlines the need for comprehensive analyses that not only document the existence of RAEs but also clarify their potential consequences for selection- and participation-related outcomes and long-term development pathways. For this reason, the present study aimed to achieve two main objectives: Part I—comprehensively quantify the prevalence and magnitude of RAEs among elite football players, considering age subgroups (i.e., youth vs. senior), contextual factors (i.e., playing positions and rank levels), and Part II—explore the potential relationship between birth quartile and selection- and participation-related outcomes (i.e., total playing time, number of matches played, and participation consistency).

## 2 Methods

Player and team data from the 2022–2023 Serie A football season were obtained from the publicly available online database Transfermarkt.^1^ Transfermarkt is an extensive, publicly accessible football database, regularly used in football analytics research. For each player, information on birthdate (day, month, and year), playing position [goalkeepers (GK), defenders (DF), midfielders (MF), and forwards (FW)], total playing time (Total Playing Time), and number of matches played (Number of Matches) was collected. A total of 789 players (Overall Cohort: mean age = 24.2 ± 5.0 years; median = 24 years), included in the official rosters of the 20 Serie A teams, were considered for analysis. The mean age (±SD) and median age for each playing position were GK: *N* = 102, 24.8 ± 6.7 years (median = 24 years), DF: *N* = 283, 24.8 ± 4.5 years (median = 24.0 years), MF: *N* = 253, 23.5 ± 4.6 years (median = 23.0 years), and FW: *N* = 151, 24.5 ± 5.1 years (median = 24.0 years). These median values were used to define the Younger and Senior subgroups within each playing position (see “Procedures” section).

### 2.1 Procedures

Players were grouped according to age category, playing position, and team ranking. For age, the Overall Cohort was divided at its median age (24 years), defining Younger (<24 years) and Senior (≥24 years) subgroups. In a second step, role-specific median ages were calculated for each playing position, and the same Younger and Senior categorization was applied within GK, DF, MF, and FW using these role-specific medians to account for positional age differences. Team ranking was defined based on the 2022–2023 final league standings as Top 1–5 (first to fifth final position), Top 6–10 (sixth to tenth), Top 11–15 (eleventh to fifteenth), and Top 16–20 (sixteenth to twentieth). RAEs were evaluated by dividing players’ birthdates into four quartiles according to the FIFA selection year (Q1 = January–March; Q2 = April–June; Q3 = July–September; Q4 = October–December).

## 3 Statistical analysis

### 3.1 Part I: relative age effects

The observed quartile distributions for each age cohort were compared to the expected quartile distributions (i.e., 25% for each quartile) using chi-squared goodness-of-fit tests (*χ*^2^). Cramer’s V (φc) was used as the effect size. The following thresholds were used: φc ≤ 0.06 trivial, 0.06 < φc ≤ 0.17 small, 0.17 < φc < 0.29 medium, and φc ≥ 0.29 large (Brustio and Boccia, 2021). Odds ratios (ORs) and 95% confidence intervals (CIs) were calculated to compare (i) players born in Q1 vs. Q4 and (ii) players born in the first half (Q1–Q2) vs. the second half (Q3–Q4) of the selection year, using Q4 as the reference category for single-quartile comparisons. Analyses were first performed for the Overall Cohort and subsequently replicated within each playing position (GK, DF, MF, and FW) and rank level (Top 1–5, Top 6–10, Top 11–15, and Top 16–20). To account for age and positional differences, both the playing position and the rank level analyses were further stratified into Younger and Senior subgroups based on their respective median ages. This approach allowed examination of RAE patterns both across the entire sample and within age-, role-, and rank-specific contexts. Finally, to quantify the proportion of non-competing players (those who did not participate in any official match, that is, 0 appearances) across birth quartiles, additional *χ*^2^ tests were performed.

### 3.2 Part II: relative age effects and selection- and participation-related outcomes

To examine associations between birth quartile (independent variable) and selection- and participation-related outcomes, one-way analysis of variance (ANOVA) tests were conducted for Total Playing Time, Number of Matches, and a Consistency Index of match participation. The Consistency Index, reflecting the regularity of match involvement, was computed as:


$$\text{Consistency Index}=\frac{\text{Number of Matches}\times \text{Total Playing Time}\;}{\text{Number}\;{\text{of Matches}}^{2}+\text{Total Playing Time}}$$

where Number of Matches denotes the total number of matches contested and Total Playing Time corresponds to the cumulative number of minutes completed over the season. The Consistency Index was calculated as a composite measure resembling a harmonic mean, but asymmetrically weighted by squaring match frequency to emphasize the regularity of participation. It reflects how evenly playing time was distributed across matches: higher values indicate consistent participation (i.e., minutes spread across many matches), whereas lower values denote irregular or concentrated exposure (e.g., a higher number of minutes in a few appearances). To the best of our knowledge, the Consistency Index has not been previously reported and is proposed here as an original composite indicator of match participation regularity.

Effect sizes were calculated using eta squared (*η*^2^). The following thresholds were used to interpret the magnitude of the effect: *η*^2^ ≤ 0.01 trivial, 0.01 < *η*^2^ ≤ 0.06 small, 0.06 < *η*^2^ ≤ 0.14 medium, and *η*^2^ > 0.14 large (Cohen, 1992). When needed, Bonferroni-corrected *post hoc* comparisons were performed to identify pairwise differences between quartiles.

Analyses were conducted for the Overall Cohort and then replicated within each playing position and rank level, with all analyses also stratified into Younger and Senior subgroups based on their specific median ages. All data were analyzed using custom-written software in MATLAB R2025a (MathWorks, Natick, MA, USA).

## 4 Results

### 4.1 Part I: relative age effect

Table 1 summarizes the relative age distribution, including chi-squared statistics and ORs (i.e., Q1 vs. Q4 and Q1–Q2 vs. Q3–Q4), for the Overall Cohort, Younger and Senior Players, and all playing positions (i.e., GK, DF, MF, FW).

**Table 1:** The relative age distribution, chi-squared, and odds ratio analyses of Serie A players according to Overall Cohort, and Younger and Senior Players.

	Total *N*	Q1%	Q2%	Q3%	Q4%	*χ* ^2^	*V*	Effect size category	OR	OR
									Q1 vs Q4	Q1–Q2 vs Q3–Q4
All playing positions										
Overall Cohort	789	36.0	23.8	21.5	18.6	55.220^***^	0.15	Small	1.9 [1.5–2.6]	1.5 [1.2, 1.8]
Younger Players	367	36.8	24.3	21.5	17.4	30.550^***^	0.17	Small	2.1 [1.4–3.2]	1.6 [1.2, 2.1]
Senior Players	422	35.3	23.5	21.6	19.7	25.020^***^	0.14	Small	1.8 [1.2–2.6]	1.4 [1.1, 1.9]
Goalkeeper										
Overall Cohort	102	43.1	22.5	24.5	9.8	22.690^***^	0.27	Medium	4.4 [1.8–10.6]	1.9 [1.1, 3.4]
Younger Players	49	36.7	20.4	32.7	10.2	8.750^**^	0.24	Medium	3.6 [1.0–12.9]	1.3 [0.6, 3.0]
Senior Players	53	49.1	24.5	17.0	9.4	19.150^***^	0.35	Large	5.2 [1.5–17.7]	2.8 [1.2, 6.3]
Defenders										
Overall Cohort	283	35.7	23.0	18.4	23.0	18.775^***^	0.15	Small	1.6 [1.0–2.4]	1.4 [1.0, 2.0]
Younger Players	121	33.9	24.0	17.4	24.8	6.767	0.14	Small	1.4 [0.7–2.7]	1.4 [0.8, 2.3]
Senior Players	162	37.0	22.2	19.1	21.6	12.732^**^	0.16	Small	1.7 [0.9–3.1]	1.5 [0.9, 2.3]
Midfielders										
Overall Cohort	253	34.4	22.5	24.9	18.2	14.302^***^	0.14	Small	1.9 [1.1–3.1]	1.3 [0.9, 1.9]
Younger Players	113	34.5	29.2	22.1	14.2	10.679^*^	0.18	Medium	2.4 [1.1–5.3]	1.8 [1.0, 3.0]
Senior Players	140	34.3	17.1	27.1	21.4	9.257^*^	0.15	Small	1.6 [0.8–3.1]	1.1 [0.7, 1.7]
Forwards										
Overall Cohort	151	34.4	28.5	19.9	17.2	11.289^***^	0.16	Small	2.0 [1.0–3.8]	1.7 [1.1, 2.7]
Younger Players	70	47.1	21.4	14.3	17.1	18.556^***^	0.30	Large	2.8 [1.1–7.0]	2.2 [1.1, 4.3]
Senior Players	81	23.5	34.6	24.7	17.3	5.050	0.14	Small	1.4 [0.5–3.4]	1.4 [0.7, 2.6]

Q1, first quartile; Q2, second quartile; Q3, third quartile; Q4, fourth quartile; *χ*^2^, chi-squared value; *V*, Cramer’s *V* effect size. OR, odds ratio and 95% confidence intervals [95% CI]; ^***^p < 0.001; ^**^p < 0.01; ^*^p < 0.05.

Data were examined by playing position (i.e., all playing position, goalkeeper, defenders, midfielders, and forwards).

**Figure 1:**
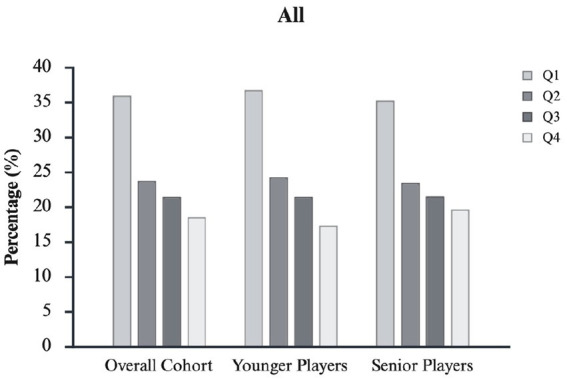
Birth-quartile distribution for the Overall Cohort and for Younger and Senior subgroups. Across all age categories, Q1 players are consistently over-represented and Q4 players under-represented relative to a uniform 25% distribution, with the most pronounced imbalance observed in Younger Players. Full descriptive statistics and inferential results are reported in Table 1.

In the Overall Cohort, birth quartile distributions were not uniform (*χ*^2^ = 55.220, *p* < 0.001, φc = 0.15, small). This pattern was consistent across all playing positions, with small-to-large effect sizes (φc range = 0.14–0.27). RAEs were the most pronounced in GK (*χ*^2^ = 22.690, *p* < 0.001, φc = 0.27, large), followed by FW (*χ*^2^ = 11.289, *p* < 0.001, φc = 0.16, small), DF (*χ*^2^ = 18.775, *p* < 0.001, φc = 0.15, small), and MF (*χ*^2^ = 14.302, *p* < 0.001, φc = 0.14, small). The OR comparison showed a disproportionate number of players born in Q1 compared with players born in Q4, with ORs ranging from 1.6 to 3.6 across playing positions. The magnitude of RAEs decreased with age: the effect tended to be stronger among Younger Players [OR = 2.1 (1.4–3.2)], while it diminished in Senior Players [OR = 1.8 (1.2–2.6)]. See Figure 1 for a visual inspection of the quartile distribution considering the Overall Cohort and Younger and Senior age groups.

**Figure 2:**
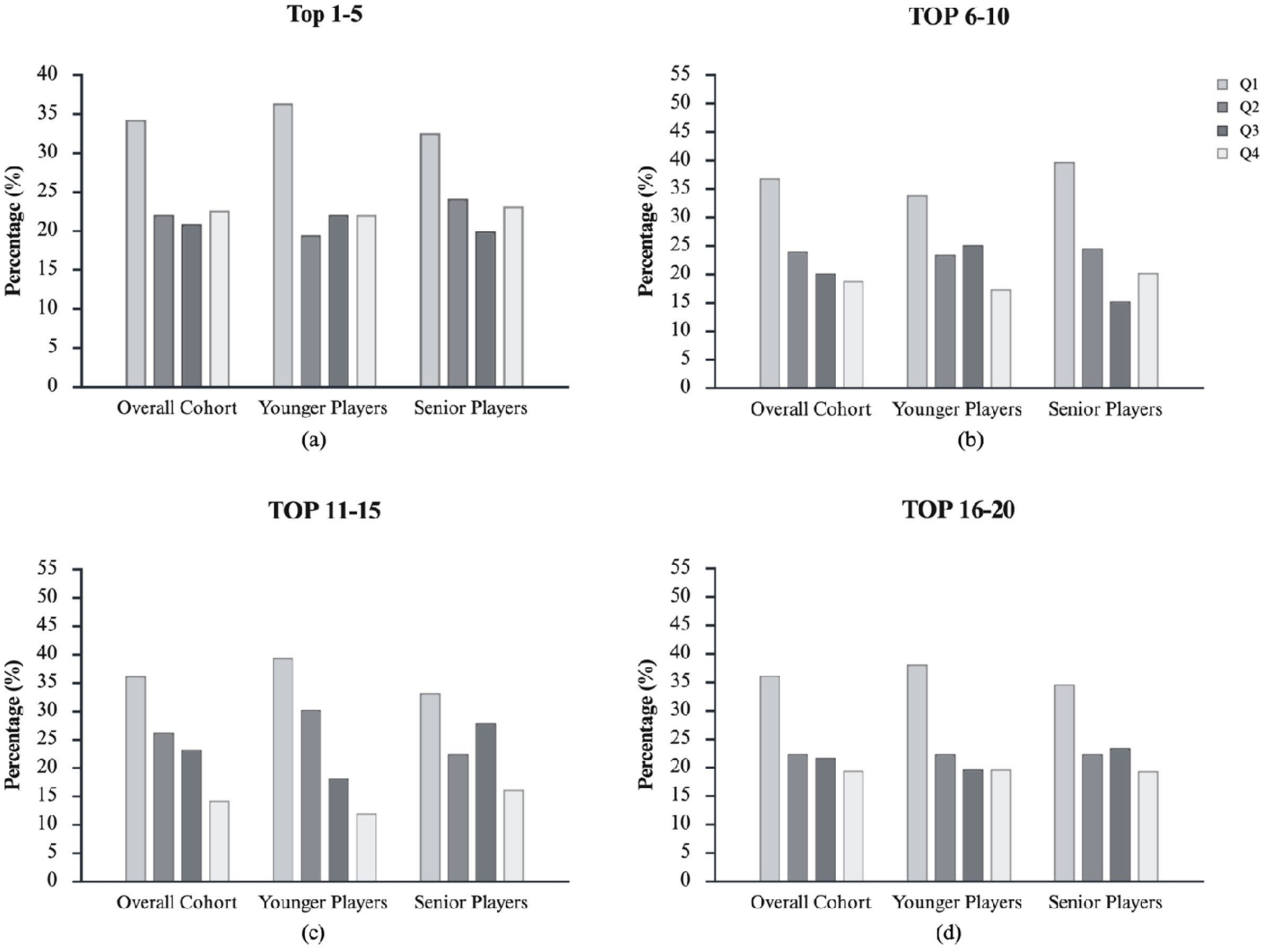
Birth-quartile distribution for the Overall Cohort and for Younger and Senior subgroups, stratified by team ranking (Top 1–5, Top 6–10, Top 11–15, Top 16–20). The over-representation of Q1 players and under-representation of Q4 players is more marked in lower-ranked teams (Top 11–15 and Top 16–20) than in top-ranked teams (Top 1–5), particularly among Younger Players. Full descriptive statistics and inferential results are reported in Table 2. Birth quartile distributions are divide in: Q1 = January–March; Q2 = April–June; Q3 = July–September; Q4 = October–December.

The analysis considers the different playing positions relevant to RAEs in the Younger GK [OR = 3.6 (1.0–12.9)], FW [OR = 2.8 (1.1–7.0)], and MF [OR = 2.4 (1.1–5.3)] players but not in DF [OR = 1.4 (0.7–2.7)]. In contrast, among Senior Players, RAEs persisted with a large effect size in GK and small effect sizes in DF and MF, and disappeared in FW. Nevertheless, for both DF and MF, no significant differences between Q1 and Q4 players were found. Figure 2 presents a visual inspection of the quartile distributions for the Overall Cohort, and Younger and Senior Players stratified by rank level.

When stratifying by rank level, birth quartile distributions were symmetrical in Top 1–5 (see Figure 2a) in the Overall Cohort (*χ*^2^ = 8.047, *p* = 0.045, φc = 0.12) and for both Younger (*χ*^2^ = 5.526, *p* = 0.137, φc = 0.12) and Senior subgroups (*χ*^2^ = 3.292, *p* = 0.349, φc = 0.11). A small but significant RAEs emerged in Top 6–10 (Figure 2b) for the Overall Cohort (*χ*^2^ = 19.052, *p* = 0.003, φc = 0.17, small), with non-significant patterns in Younger (*χ*^2^ = 6.379, *p* = 0.095, φc = 0.14) and a medium effect in Senior subgroups (*χ*^2^ = 15.667, *p* = 0.001, φc = 0.21). The effect strengthened in Top 11–15 (Figure 2c), significant overall (*χ*^2^ = 20.340, *p* = 0.001, φc = 0.18, medium), driven by Younger (*χ*^2^ = 17.560, *p* < 0.001, φc = 0.24, medium) but not Senior subgroups (*χ*^2^ = 7.107, *p* = 0.069, φc = 0.15, small). Corresponding Q1 vs. Q4 ORs were 2.5 [1.4–4.5] for the Overall Cohort, 3.3 [1.4–7.6] in Younger, and 2.1 [1.0–4.4] in Senior subgroups. In Top 16–20 (Figure 2d), a small overall effect persisted (*χ*^2^ = 11.864, *p* = 0.008, φc = 0.15, small), with non-significant trends in Younger (*χ*^2^ = 11.864, *p* = 0.067, φc = 0.18, medium) and Senior subgroups (*χ*^2^ = 5.200, *p* = 0.158, φc = 0.13, small). The overall Q1 vs. Q4 OR was 1.9 [1.0–3.3] and Q1–Q2 vs. Q3–Q4 OR 1.4 [0.9–2.2]. For more details about quartile distributions, chi-squared statistics, and ORs, see Table 2.

**Table 2:** The relative age distribution, chi-squared and odds ratio analyses of Serie A players according to Overall Cohort, Younger and Senior Players.

	Total *N*	Q1%	Q2%	Q3%	Q4%	*χ* ^2^	*V*	Effect size category	OR	OR
									Q1 vs Q4	Q1–Q2 vs Q3–Q4
Top 1–5										
Overall Cohort	172	34.3	22.1	20.9	22.7	8.047^*^	0.12	Small	1.5 [0.8–2.7]	1.3 [0.8–2.0]
Younger Players	77	36.4	19.5	22.1	22.1	5.526	0.15	Small	1.6 [0.7–4.0]	1.3 [0.7–2.4]
Senior Players	95	32.6	24.2	20.0	23.2	3.292	0.11	Small	1.4 [0.6–3.1]	1.3 [0.7–2.3]
Top 6–10										
Overall Cohort	233	36.9	24.0	20.2	18.9	19.052^***^	0.17	Small	2.0 [1.2–3.3]	1.6 [1.1–2.3]
Younger Players	115	33.9	23.5	25.2	17.4	6.379	0.14	Small	2.0 [0.9–4.1]	1.3 [0.8–2.3]
Senior Players	118	39.8	24.6	15.3	20.3	15.667^**^	0.21	Medium	2.0 [1.0–4.0]	1.8 [1.1–3.0]
Top 11–15										
Overall Cohort	210	36.2	26.2	23.3	14.3	20.340^***^	0.18	Medium	2.5 [1.4–4.5]	1.7 [1.1–2.4]
Younger Players	99	39.4	30.3	18.2	12.1	17.560^**^	0.24	Medium	3.3 [1.4–7.6]	2.3 [1.3–4.1]
Senior Players	111	33.3	22.5	27.9	16.2	7.107	0.15	Small	2.1 [1.0–4.4]	1.3 [0.7–2.1]
Top 16–20										
Overall Cohort	174	36.2	22.4	21.8	19.5	11.864^**^	0.15	Small	1.9 [1.0–3.3]	1.4 [0.9–2.2]
Younger Players	76	38.2	22.4	19.7	19.7	11.864	0.18	Medium	1.9 [0.8–4.7]	1.5 [0.8–2.9]
Senior Players	98	34.7	22.4	23.5	19.4	5.200	0.13	Small	1.8 [0.8–3.9]	1.3 [0.8–2.3]

Q1, first quartile; Q2, second quartile; Q3, third quartile; Q4, fourth quartile; *χ*^2^, chi-squared value; *V*, Cramer’s *V* effect size. OR, odds ratio, and 95% confidence intervals [95% CI]; ^***^p < 0.001; ^**^p < 0.01; ^*^p < 0.05.

Data were examined according to rank level [i.e., Top 1–5 (first to fifth placing), Top 6–10 (sixth to tenth placing), Top 11–15 (eleventh to fifteenth placing), and Top 16–20 (sixteenth to twentieth placing)].

**Figure 3:**
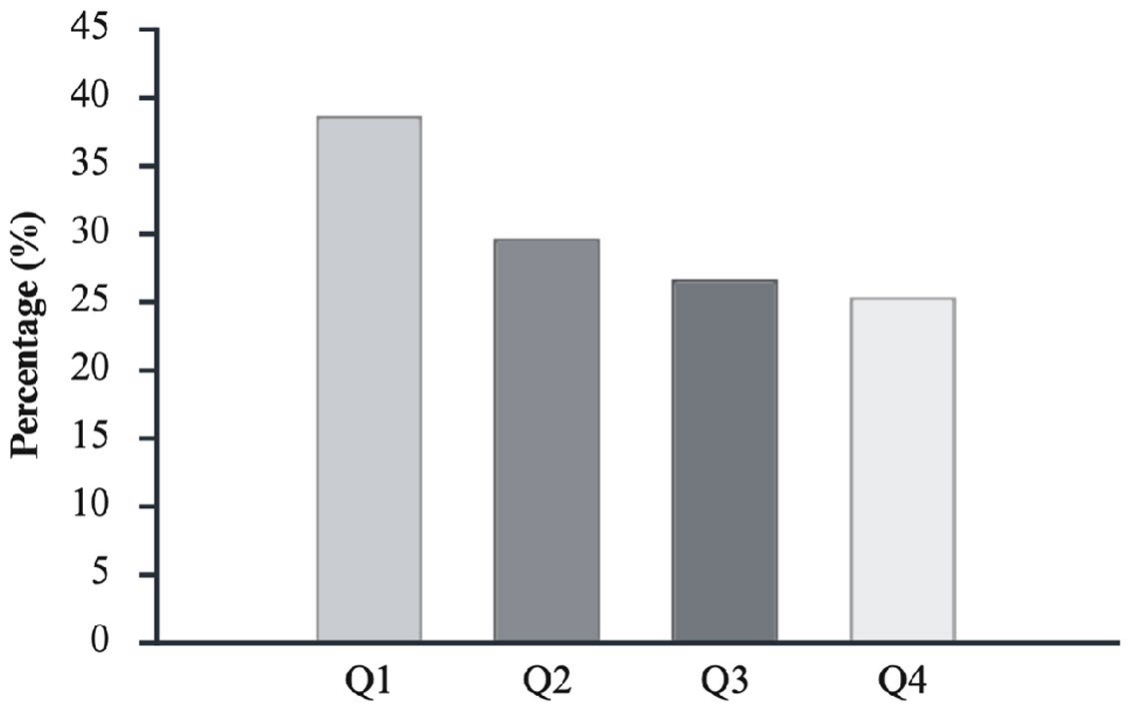
Percentage distribution across birth quartiles (Q1–Q4) for non-competing players (0 official match appearances) in the Overall Cohort.

Figure 3 shows the proportion of non-competing players across birth quartiles. Birth quartile distributions were not uniform (*χ*^2^ = 10.898, *p* < 0.05, φc = 0.11, small). The OR comparison showed a disproportionate number of those born in Q1 compared with players born in Q4, with an OR of 2.7 [1.4, 5.0].

### 4.2 Part II: relative age effect and selection- and participation-related outcomes

**Figure 4:**
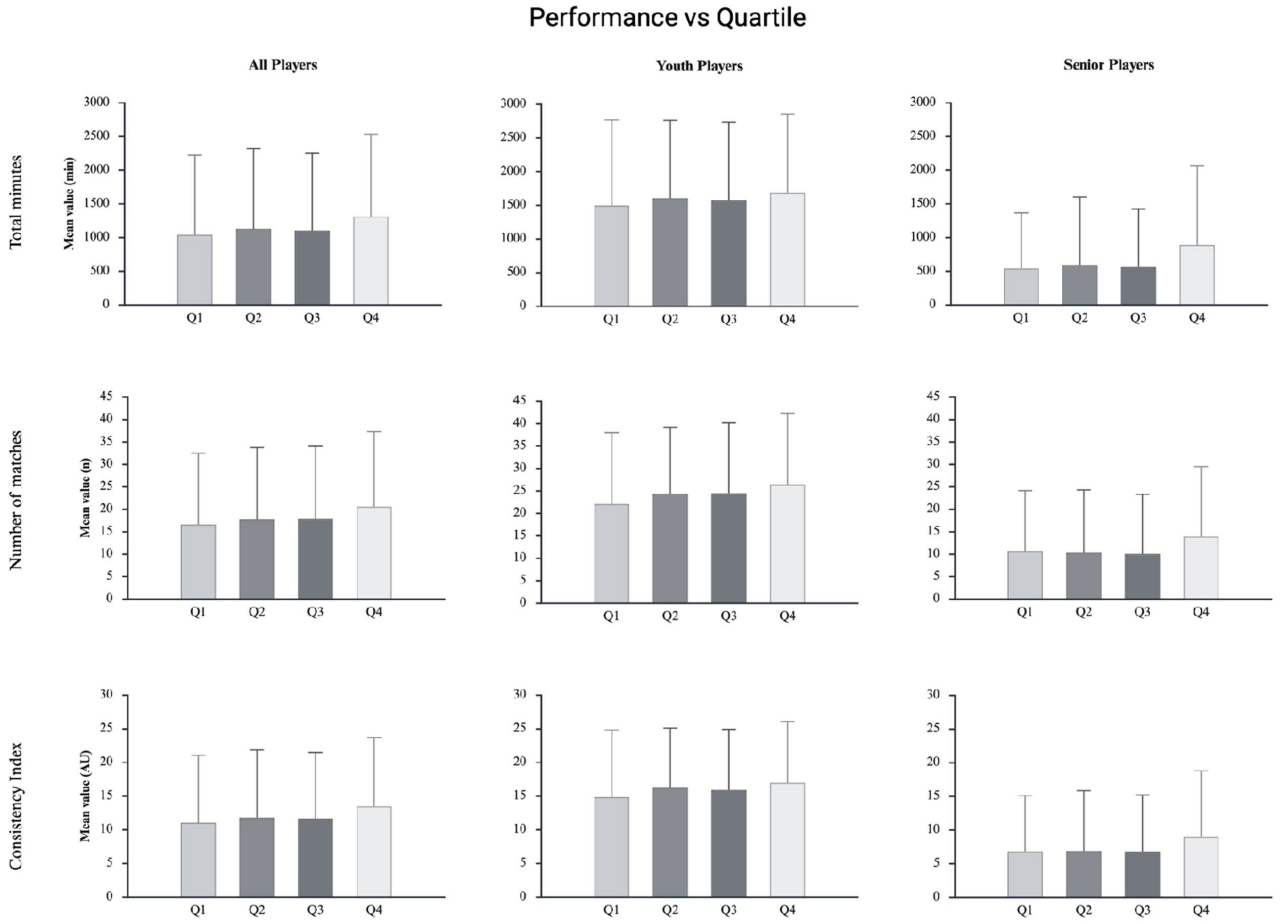
Mean and standard deviation of performance-related outcomes (i.e., total playing time, number of matches played, and consistency index) across birth quartiles, reported separately for Overall Cohort, Younger and Senior Players.

Figure 4 shows the relationship between birth quartiles and selection- and participation-related outcomes (i.e., Total Playing Time, Number of Matches, and Consistency Index) for the Overall Cohort and for the Younger and Senior subgroups.

In the Overall Cohort, although mean values for Total Playing Time, Number of Matches, and Consistency Index suggested a slight increasing trend from Q1 to Q4, the analyses did not reveal any statistically significant effects of birth quartile on selection- and participation-related outcomes. Total Playing Time ranged from 1,042.8 ± 1,181.1 (Q1) to 1,314.0 ± 1,223.0 min (Q4). However, one-way ANOVA indicated no significant effect of birth quartile on Total Playing Time (*F* = 1.72, *p* = 0.162, *η*^2^ = 0.006). Similarly, no significant differences were found in the Number of Matches across quartiles, which ranged from 16.7 ± 15.8 (Q1) to 20.6 ± 16.7 (Q4) (*F* = 1.95, *p* = 0.119, *η*^2^ = 0.007). For the Consistency Index, mean values showed a slight increase from Q1 (11.06 ± 10.02) to Q4 (13.50 ± 10.20), but no statistically significant differences were observed across birth quartiles (*F* = 1.95, *p* = 0.120, *η*^2^ = 0.007). Subgroup analyses by age revealed similar results. Among Younger Players, Total Playing Time ranged from 541.8 ± 826.5 (Q1) to 835.2 ± 1,128.5 min (Q4), Number of Matches ranged from 10.1 ± 13.3 (Q3) to 13.0 ± 14.8 (Q4), and Consistency Index ranged from 6.85 ± 8.29 (Q1) to 8.96 ± 9.83 (Q4). None of these differences were statistically significant (all *p* > 0.05). Among Senior Players, Total Playing Time ranged from 1496.8 ± 1269.8 (Q1) to 1683.2 ± 1169.5 min (Q4), Number of Matches ranged from 22.1 ± 15.9 (Q1) to 26.5 ± 15.7 (Q4), and the Consistency Index ranged from 14.90 ± 9.96 (Q1) to 17.00 ± 9.10 (Q4). ANOVA revealed no significant main effect of birth quartile on Total Playing Time (Younger: *F* = 1.225, *p* = 0.157, *η*^2^ = 0.012; Senior: *F* = 0.584, *η*^2^ = 0.005), Number of Matches (Younger: *F* = 0.374, *η*^2^ = 0.004; Senior: *F* = 1.221, *η*^2^ = 0.010), or Consistency Index (Younger: *F* = 0.720, *η*^2^ = 0.007; Senior: *F* = 0.803, *η*^2^ = 0.007).

Overall, no statistically significant differences were observed in any selection- or participation-related outcomes across birth quartiles (all *p* > 0.05). The same patterns were observed across different playing positions and rank levels. For more information, see Supplementary Material S1.

## 5 Discussion

This study investigated the prevalence and magnitude of RAEs among elite football players across age-related subgroups, playing positions, and rank levels (Part I), and explored the potential relationship between birth quartile and selection- and participation-related outcomes (Part II). In Part I, we observed skewed birthdate distributions in Serie A rosters favoring relatively older players [OR = 1.8 (1.3, 2.4)]. This pattern was evident both in the Overall Cohort and when players were stratified by age, with small effect sizes [OR = 2.0 (1.3, 3.1) for Younger and OR = 2.0 (1.3, 3.1) for Senior]. Although heterogeneous, RAEs were also evident when analyzing different playing positions. Indeed, although RAEs were apparent in the Overall Cohort, they emerged at the youth level primarily in GK [OR = 4.4 (1.8–10.6)] followed by FW [OR = 2.0 (1.0–3.8)], MF [OR = 1.9 (1.1–3.1)], and DF [OR = 1.6 (1.0–2.4)] with small-to-large effect sizes, and only among GK and DF in the Senior category (large and small effect sizes, respectively). In parallel, RAEs seemed to be more pronounced in lower ranked contexts (i.e., Top 11–15 and Top 16–20) than in the higher-ranked ones (Top 1–5 and Top 6–10). In Part II, although no statistically significant differences emerged, small trends were observed, suggesting slightly higher values for selection- and participation-related outcomes in players born in later quartiles (especially Q4 players). Moreover, within the Overall Cohort, the proportion of non-competing players was roughly three times higher among Q1 players than among Q4 players.

RAE analysis showed that, regardless of age, there were consistent asymmetries in the quartile distribution, with Q1 players being overrepresented compared to Q4 equivalents (general mean: 36.0% in Q1 vs. 19.7% in Q4). The probability of being included in the rosters for Q1 players was 1.9 [1.5, 2.6] times higher in comparison with Q4 players. These results were in line with other studies in the same cultural context, suggesting that RAEs also persisted in the Italian elite football league (Brustio et al., 2018; Lupo et al., 2019; Morganti et al., 2023). Thus, we observed a residual selection bias in the Italian context, indicating knock-on effects in which the overrepresentation of relatively older players during youth persists into senior levels. Nevertheless, it is necessary to point out that the RAEs demonstrated only a small effect size here compared with a medium effect size when the level of competition increases (i.e., players selected for national teams) (Brustio et al., 2024).

RAE trends were also evident when the sample was stratified by age. Our results suggested that the effect was more pronounced in the Younger than in the Senior subgroup (Cramér’s *V* = 0.17 vs. 0.14), confirming, even in an elite football context, that age modulates the magnitude of RAEs and that it decreases with increasing age (Bozděch et al., 2023). In other words, selection biases associated with relative age appear firmer in earlier career stages and may attenuate over time. Furthermore, positional analyses revealed that RAEs were present across all playing roles, with FW and GK in the Younger subgroup exhibiting the most considerable asymmetries (medium-to-large effect sizes), thus confirming the data observed in the youth Italian national context (Brustio et al., 2024). In the Senior group, the effect was detectable mainly among GK (large effect size), DF, and MF (small effect sizes). These results support the notion that RAEs may be more influential in roles that demand high physical and tactical maturity at early stages. At the same time, their impact becomes less consistent as players advance in their careers.

Interestingly, the analysis by rank level suggested that the magnitude of RAEs was higher in lower ranked teams than in top-ranked teams. Indeed, although it is well known that RAEs tend to increase at higher competitive levels (Cobley et al., 2009; Bozděch et al., 2023), our results suggested a different trend, with larger RAEs in lower ranked teams. This discrepancy likely reflects study design: we modelled ranking within the same elite league, whereas much of the literature compares across tiers. Thus, residual selection asymmetries appear concentrated in lower ranked squads.

Despite the evident selection bias, no statistically significant differences emerged between quartiles in Total Playing Time, Number of Matches, or Consistency Index. Overall, although descriptive statistics suggested a slight increase in performance metrics from Q1 to Q4, the analyses did not support a statistically significant relationship between birth quartile and either match involvement or participation consistency. This pattern is also visible when stratified by age. Thus, within the elite level of Serie A football, the RAEs do not manifest in terms of accumulated playing time, match count, or regularity of match exposure. Our results align with previous evidence in youth cohorts (Vaeyens et al., 2005; Sæther, 2016) showing no direct relationship between birth quartile and match-related outcomes. In other words, our results suggest that while there is a residual selection bias (i.e., knock-on effects) in birth quartile distribution at the elite level (i.e., higher odds of being selected for Q1 players), this bias does not translate into differences in selection- and participation-related outcomes.

Nevertheless, the slight descriptive increase from Q1 to Q4 is consistent with the hypothesis that relatively Younger Players who successfully reach the elite level may constitute a positively selected subgroup, capable of matching or even surpassing the participation profiles of their relatively older peers. Notably, when examining non-competing players, a substantial proportion belonged to the first quartile. This finding suggests that although Q1 players are more frequently selected, such selection does not necessarily result in greater competitive participation. This scenario is consistent with the so-called *underdog hypothesis* (McCarthy et al., 2016; Gibbs et al., 2012). According to this view, relatively Younger Players who overcome early selection disadvantages may develop compensatory advantages (e.g., in psychological resilience, tactical adaptability, or technical efficiency) that enable them to compete on equal or even superior terms at the senior level, despite being under-represented in earlier stages of the pathway. The slight, non-significant trend toward higher selection- and participation-related outcomes among Q4 players observed in the present study can therefore be interpreted as preliminary, descriptive support for this hypothesis. Taken together with the over-representation of Q1 players in rosters and among non-competing players, these findings indicate that RAEs appear to operate primarily at the level of squad selection (i.e., who gains access to the professional roster and who remains a non-competing vs. competing player), rather than at the level of match selection (number of appearances) and participation-related outcomes (total playing time and participation consistency). This apparent paradox appears to be supported by a recent study by Bezuglov et al. (2023), which also demonstrated that relatively older football players are valued similarly in terms of market value. Consequently, further analyses could be promoted to systematically verify whether clear and positive relationships exist between players’ market value and seasonal playing time and number of matches, including, but not limited to, birth quartile classification. In addition, in the present study, no analyses have been applied to discriminate between the more and less important matches performed by players. Also, for this reason, further research could investigate whether, despite equality in the number of matches and playing time among players, relatively older athletes actually participate more often in important matches. In contrast, relatively Younger Players mostly appear in less important ones.

Despite these results, it is necessary to point out some limitations. The Transfermarkt database provides extensive coverage for soccer data, but it is not an official league database. Moreover, the present study analyzed RAEs in professional football teams in Italy. Nevertheless, according to data from the Transfermarkt database, during the 2022–2023 season, 59% of players were foreign-born (teams’ average of 59 ± 12%; minimum = 32%; maximum = 80%). Thus, professional players competing in Italy have been developed within other national systems. Consequently, since RAEs are primarily evident during youth talent development phases, the RAE patterns observed at the professional level likely represent residual outcomes of those earlier selection and development processes. Therefore, it cannot be assumed that the RAEs detected in Italian professional teams directly reflect biases within the Italian talent development pathway. This heterogeneity limits the extent to which national-level interpretations of RAEs can be generalized from the present findings. A further limitation concerns the goalkeeper subgroup. Goalkeepers were included as a separate positional category; however, the number of players in this role is necessarily small at the team level, which substantially reduces statistical power when stratifying by age category, rank level, and birth quartile. As a result, RAE estimates for goalkeepers should be interpreted with caution, and no firm conclusions can be drawn for this specific position. Future studies specifically designed for goalkeepers, possibly aggregating data across multiple seasons or leagues, are needed to provide more robust evidence on RAEs in this role. It should be noted that the variables analyzed (i.e., playing time, number of matches, and Consistency Index) reflect selection and participation opportunities rather than athletic performance *per se*. Therefore, the absence of differences across quartiles does not imply equivalent performance, but rather similar utilization patterns among selected players. In addition, these indicators may be affected by factors unrelated to performance, such as injuries, illnesses, suspensions, or tactical rotation, which can limit playing time or match participation independently of a player’s performance level. Finally, it should be acknowledged that the Consistency Index of match participation used in this study is an original operationalization and has not yet been validated against other indicators of player utilization or performance. Although it was explicitly designed to minimize the confounding effect of substitute appearances by capturing the regularity of match involvement, its psychometric and practical properties remain to be confirmed. Future research should therefore examine the robustness of this index, compare it with alternative measures of participation (e.g., match-to-match variability in minutes played and availability indices), and evaluate its usefulness in different competitive contexts and across multiple seasons.

In conclusion, while clear RAEs were evident in the Italian elite football context, these asymmetries did not translate into meaningful differences in competitive participation at the match level. Specifically, Serie A rosters showed residual selection asymmetries, favoring relatively older players across both Younger and Senior subgroups, and with playing positional heterogeneity. Notably, when ranking was operationalized intra-league, RAE magnitudes were higher in lower ranked teams than in top-ranked teams, indicating that selection biases may be more salient in squads with lower talent pools.

RAEs persist in Serie A rosters but appear to operate primarily as a selection-level bias within talent development and squad selection, affecting which players reach and remain at the elite level, rather than influencing coaches’ decisions regarding match participation once players are part of the professional environment.

## Data Availability

The original contributions presented in the study are included in the article/Supplementary material, further inquiries can be directed to the corresponding author.

## Footnotes

1
https://www.transfermarkt.it
