# Supplementary material for: Relative age selection bias does not translate into a playing-time advantage: evidence from Italian Serie A football
Source: Front Psychol. 2026 Jan 5;16:1733277. doi: 10.3389/fpsyg.2025.1733277 (PMC12812947; doi:10.3389/fpsyg.2025.1733277)
Supplement: Supplementary file 1 [file Table_1.DOCX]

| **Supplementary Material 1.** Mean standard deviation of performance-related outcomes and ANOVA outcomes (Total Playing Time Number of Matches played and Consistency Index) across birth quartiles reported separately for all players the Younger subgroup and the Senior subgroup. | | | | | | | | | | | | | | | |
| --- | --- | --- | --- | --- | --- | --- | --- | --- | --- | --- | --- | --- | --- | --- | --- |
|  | Total Playing Time | | | | | Number of Matches | | | | | Consistency Index | | | | |
|  | Q1 | Q2 | Q3 | Q4 | ANOVA outcomes | Q1 | Q2 | Q3 | Q4 | ANOVA outcomes | Q1 | Q2 | Q3 | Q4 | ANOVA outcomes |
| **All Playing Positions** | | | | | | | | | | | | | | | |
| Overall Cohort | 1042.8±1181.1 | 1128.7±1195.2 | 1104.2 ± 1146.1 | 1314.0 ± 1223.0 | F = 1.719 η² = 0.007 | 16.7 ± 15.8 | 17.8 ± 16.0 | 17.8 ± 16.2 | 20.6 ± 16.7 | F = 1.954 η² = 0.007 | 11.1 ± 10.0 | 11.8 ± 10.0 | 11.7 ± 9.8 | 13.5 ± 10.2 | F = 1.951 η² = 0.007 |
| Younger Players | 541.8±826.5 | 595.9±1007.2 | 563.4 ± 859.6 | 835.2 ± 1128.5 | F = 1.543 η² = 0.013 | 10.7 ± 13.5 | 10.3 ± 14.0 | 10.1 ± 13.3 | 13.0 ± 14.8 | F = 0.637 η² = 0.005 | 6.8 ± 8.3 | 6.9 ± 9.0 | 6.8 ± 8.3 | 9.0 ± 9.8 | F = 1.024 η² = 0.008 |
| Senior Players | 1496.8±1269.8 | 1607.6±1151.3 | 1573.7 ± 1160.5 | 1683.2 ± 1169.5 | F = 0.460 η² = 0.003 | 22.1 ± 15.9 | 24.4 ± 14.8 | 24.5 ± 15.6 | 26.5 ± 15.7 | F = 1.519 η² = 0.011 | 14.9 ± 10.0 | 16.3 ± 8.9 | 15.9 ± 9.0 | 17.0 ± 9.1 | F = 1.044 η² = 0.007 |
| **Playing Positions** | | | | | | | | | | | | | | | |
| **Goalkeepers** |  |  |  |  |  |  |  |  |  |  |  |  |  |  |  |
| Overall Cohort | 884.0 ± 1481.9 | 908.9 ± 1382.0 | 642.8 ± 1092.4 | 533.8 ± 977.4 | F = 0.359 η² = 0.011 | 10.0 ± 16.4 | 10.7 ± 16.5 | 7.3 ± 12.2 | 6.0 ± 10.9 | F = 0.398 η² = 0.012 | 7.2 ± 11.1 | 7.6 ± 11.0 | 5.6 ± 9.0 | 4.8 ± 8.3 | F = 0.293 η² = 0.009 |
| Younger Players | 49.4 ± 115.8 | 0.0 ± 0.0 | 208.4 ± 636.2 | 0.0 ± 0.0 | F = 0.893 η² = 0.056 | 0.9 ± 1.8 | 0.0 ± 0.0 | 2.3 ± 7.0 | 0.0 ± 0.0 | F = 0.809 η² = 0.051 | 0.7 ± 1.6 | 0.0 ± 0.0 | 1.9 ± 5.4 | 0.0 ± 0.0 | F = 0.886 η² = 0.056 |
| Senior Players | 1461.9 ± 1708.6 | 1608.1 ± 1511.2 | 1415.1 ± 1331.4 | 1067.6 ± 1198.9 | F = 0.145 η² = 0.009 | 16.3 ± 18.9 | 18.8 ± 18.2 | 16.1 ± 14.7 | 12.0 ± 13.3 | F = 0.186 η² = 0.011 | 11.6 ± 12.7 | 13.5 ± 11.7 | 12.3 ± 10.6 | 9.5 ± 9.9 | F = 0.147 η² = 0.009 |
| **Defenders** | | | | | | | | | | | | | |  |  |
| Overall Cohort | 1071.8±1163.2 | 1314.5±1167.8 | 1304.2±1272.4 | 1569.5±1302.1 | F=2.232 η²=0.023 | 15.7±15.0 | 18.8±15.0 | 18.8±16.0 | 22.8±16.7 | F=2.777 η²=0.029 | 13.9±9.1 | 15.9±8.4 | 16.0±8.7 | 18.1±8.7 | F=2.410 η²=0.031 |
| Younger Players | 739.6±1026.3 | 950.2±1223.0 | 683.6±940.9 | 1207.4±1302.3 | F=1.278 η²=0.032 | 11.2±13.5 | 13.6±15.5 | 11.4±13.5 | 17.1±16.4 | F=1.061 η²=0.026 | 12.7±8.6 | 14.6±9.3 | 12.8±8.2 | 16.2±9.3 | F=0.749 η²=0.029 |
| Senior Players | 1298.7±1204.2 | 1608.1±1048.1 | 1724.5±1307.6 | 1879.9±1237.0 | F=1.985 η²=0.036 | 18.7±15.3 | 23.0±13.4 | 23.7±15.8 | 27.6±15.7 | F=2.730 η²=0.049 | 14.6±9.4 | 16.6±7.8 | 17.4±8.6 | 19.3±8.3 | F=2.142 η²=0.042 |
| **Midfielders** | | | | | | | | | | | | | | | |
| Overall Cohort | 1107.6±1146.6 | 968.9±1238.2 | 1039.6±1056.0 | 1153.3±1148.4 | F=0.274 η²=0.003 | 19.3±15.8 | 16.1±16.1 | 18.4±15.8 | 19.4±15.7 | F=0.539 η²=0.006 | 15.8±8.1 | 13.4±9.5 | 13.7±8.7 | 14.6±8.8 | F=0.896 η²=0.013 |
| Younger Players | 461.5±670.8 | 325.9±723.5 | 589.1±965.0 | 339.3±549.5 | F=0.694 η²=0.019 | 10.7±13.0 | 7.4±11.3 | 10.9±14.2 | 7.0±8.3 | F=0.781 η²=0.021 | 11.2±6.9 | 7.6±7.4 | 9.1±8.9 | 8.4±5.8 | F=0.891 η²=0.037 |
| Senior Players | 1632.5±1189.2 | 1853.0±1263.2 | 1335.9±1018.5 | 1587.4±1153.2 | F=1.054 η²=0.023 | 26.3±14.4 | 28.2±13.9 | 23.4±15.0 | 26.0±14.7 | F=0.592 η²=0.013 | 18.2±7.7 | 18.5±8.3 | 16.3±7.6 | 16.6±8.7 | F=0.587 η²=0.013 |
| **Forwards** | | | | | | | | | | | | | | | |
| Overall Cohort | 1012.7±993.6 | 1177.0±1058.7 | 1277.8±1068.3 | 1259.8±1101.2 | F=0.552 η²=0.011 | 19.8±15.6 | 22.0±16.2 | 23.7±17.2 | 23.0±17.8 | F=0.435 η²=0.009 | 15.0±7.9 | 15.8±8.2 | 15.8±8.5 | 15.7±9.4 | F=0.070 η²=0.002 |
| Younger Players | 631.6±875.7 | 700.1±1014.6 | 442.6±703.2 | 795.9±1083.1 | F=0.287 η²=0.013 | 14.2±15.3 | 14.4±15.8 | 10.4±15.6 | 14.1±15.2 | F=0.175 η²=0.008 | 12.1±8.2 | 12.2±8.8 | 8.8±8.4 | 12.3±9.8 | F=0.293 η²=0.019 |
| Senior Players | 1674.6±839.7 | 1432.5±1007.9 | 1695.5±978.2 | 1657.4±985.1 | F=0.404 η²=0.015 | 29.6±10.5 | 26.1±15.1 | 30.4±14.0 | 30.6±16.7 | F=0.505 η²=0.019 | 18.6±5.8 | 17.3±7.5 | 18.2±7.3 | 17.9±8.7 | F=0.126 η²=0.005 |
| **Rank Level** | | | | | | | | | | | | | | | |
| **Top 1-5** | | | | | | | | | | | | | | | |
| Overall Cohort | 1320.8±1389.9 | 1486.6±1328.3 | 1312.0±1206.5 | 1499.0±1461.3 | F=0.238 η²=0.004 | 20.5±18.5 | 24.3±18.3 | 23.9±19.4 | 22.9±18.8 | F=0.418 η²=0.007 | 12.9±11.4 | 15.0±10.8 | 13.9±10.6 | 14.5±11.6 | F=0.305 η²=0.005 |
| Younger Players | 378.5±749.1 | 1189.5±1543.0 | 702.4±913.4 | 492.7±895.3 | F=2.241 η²=0.084 | 9.5±14.3 | 19.7±20.3 | 13.4±16.9 | 8.6±12.0 | F=1.746 η²=0.067 | 5.3±7.9 | 11.9±12.2 | 8.3±9.7 | 6.1±8.2 | F=1.805 η²=0.069 |
| Senior Players | 2172.0±1285.5 | 1680.3±1163.2 | 1857.4±1193.1 | 2276.5±1341.7 | F=1.130 η²=0.036 | 30.4±16.2 | 27.3±16.6 | 33.3±16.6 | 34.0±15.4 | F=0.795 η²=0.026 | 19.8±9.5 | 17.0±9.5 | 18.9±8.9 | 20.9±9.5 | F=0.722 η²=0.023 |
| **Top 6-10** | | | | | | | | | | | | | | | |
| Overall Cohort | 1235.9±1276.5 | 1341.3±1383.0 | 904.5±1216.4 | 1498.7±1309.7 | F=1.744 η²=0.022 | 19.4±16.9 | 19.8±17.6 | 15.1±17.1 | 23.9±18.6 | F=1.942 η²=0.025 | 12.6±10.6 | 13.1±11.1 | 9.8±10.2 | 15.0±10.8 | F=1.858 η²=0.024 |
| Younger Players | 586.8±937.5 | 431.4±858.4 | 464.7±748.4 | 993.1±1367.9 | F=1.563 η²=0.041 | 10.7±14.9 | 7.7±12.4 | 8.8±12.1 | 15.0±17.3 | F=1.129 η²=0.030 | 6.8±9.0 | 5.2±8.2 | 6.0±7.8 | 10.0±11.1 | F=1.234 η²=0.032 |
| Senior Players | 1774.6±1277.1 | 2188.4±1237.4 | 1613.1±1489.9 | 1920.0±1119.6 | F=0.948 η²=0.024 | 26.6±15.2 | 30.9±14.0 | 25.3±19.3 | 31.4±16.5 | F=0.977 η²=0.025 | 17.4±9.5 | 20.4±8.0 | 15.8±10.9 | 19.1±8.9 | F=1.123 η²=0.029 |
| **Top 11-15** | | | | | | | | | | | | | | | |
| Overall Cohort | 858.1±1003.8 | 801.4±819.9 | 1014.8±1028.1 | 1197.3±1036.7 | F=1.346 η²=0.019 | 14.0±13.2 | 13.8±12.2 | 15.5±13.5 | 17.7±13.5 | F=0.728 η²=0.010 | 9.8±8.8 | 9.7±8.1 | 10.9±9.1 | 12.6±9.3 | F=0.938 η²=0.013 |
| Younger Players | 597.7±778.3 | 497.8±660.5 | 370.7±751.5 | 1020.7±1082.4 | F=1.815 η²=0.054 | 11.4±11.8 | 9.4±10.7 | 7.1±11.4 | 15.0±14.4 | F=1.252 η²=0.038 | 7.8±7.8 | 6.6±7.2 | 4.9±7.3 | 10.6±10.1 | F=1.430 η²=0.043 |
| Senior Players | 1132.6±1144.0 | 1165.6±855.5 | 1388.7±988.7 | 1315.1±1019.2 | F=0.432 η²=0.012 | 16.8±14.2 | 19.2±11.8 | 20.3±12.3 | 19.5±12.9 | F=0.475 η²=0.013 | 11.8±9.5 | 13.3±7.7 | 14.4±8.3 | 13.9±8.8 | F=0.549 η²=0.015 |
| **Top 16-2**0 | | | | | | | | | | | | | | | |
| Overall Cohort | 741.7±921.0 | 936.1±1095.9 | 1269.7±1131.9 | 965.9±869.4 | F=2.202 η²=0.037 | 12.6±13.0 | 14.0±14.0 | 18.4±13.9 | 16.3±12.9 | F=1.681 η²=0.029 | 8.7±8.6 | 10.0±9.4 | 13.1±9.1 | 11.3±8.2 | F=2.094 η²=0.036 |
| Younger Players | 563.5±824.0 | 506.2±1051.8 | 828.2±1091.3 | 864.6±1070.6 | F=0.596 η²=0.024 | 10.8±13.4 | 7.9±12.4 | 12.5±12.8 | 13.7±14.8 | F=0.586 η²=0.024 | 7.1±8.5 | 5.7±8.7 | 9.2±9.0 | 9.5±9.9 | F=0.682 η²=0.028 |
| Senior Players | 893.7±982.7 | 1268.3±1032.0 | 1557.7±1084.9 | 1045.9±692.1 | F=2.313 η²=0.069 | 14.1±12.8 | 18.8±13.5 | 22.3±13.5 | 18.3±11.1 | F=1.946 η²=0.058 | 10.1±8.6 | 13.3±8.7 | 15.6±8.4 | 12.7±6.6 | F=2.164 η²=0.065 |
